# Computational Analysis of Flow Structures in Turbulent Ventricular Blood Flow Associated With Mitral Valve Intervention

**DOI:** 10.3389/fphys.2022.806534

**Published:** 2022-06-30

**Authors:** Joel Kronborg, Frida Svelander, Samuel Eriksson-Lidbrink, Ludvig Lindström, Carme Homs-Pons, Didier Lucor, Johan Hoffman

**Affiliations:** ^1^ Department of Computational Science and Technology, School of Electrical Engineering and Computer Science, KTH Royal Institute of Technology, Stockholm, Sweden; ^2^ Laboratoire Interdisciplinaire des Sciences du Numérique (LISN), CNRS, Université Paris-Saclay, Orsay, France

**Keywords:** patient-specific heart modelling, left ventricle, mitral valve clip, finite element method, FEM, turbulent blood flow, triple decomposition of velocity gradient tensor

## Abstract

Cardiac disease and clinical intervention may both lead to an increased risk for thrombosis events due to a modified blood flow in the heart, and thereby a change in the mechanical stimuli of blood cells passing through the chambers of the heart. Specifically, the degree of platelet activation is influenced by the level and type of mechanical stresses in the blood flow. In this article we analyze the blood flow in the left ventricle of the heart through a computational model constructed from patient-specific data. The blood flow in the ventricle is modelled by the Navier-Stokes equations, and the flow through the mitral valve by a parameterized model which represents the projected opening of the valve. A finite element method is used to solve the equations, from which a simulation of the velocity and pressure of the blood flow is constructed. The intraventricular blood flow is complex, in particular in diastole when the inflow jet from the atrium breaks down into turbulent flow on a range of scales. A triple decomposition of the velocity gradient tensor is then used to distinguish between rigid body rotational flow, irrotational straining flow, and shear flow. The triple decomposition enables the separation of three fundamentally different flow structures, that each generates a distinct type of mechanical stimulus on the blood cells in the flow. We compare the results in a simulation where a mitral valve clip intervention is modelled, which leads to a significant modification of the intraventricular flow. Further, we perform a sensitivity study of the results with respect to the positioning of the clip. It was found that the shear in the simulation cases treated with clips increased more compared to the untreated case than the rotation and strain did. A decrease in valve opening area of 64% in one of the cases led to a 90% increase in rotation and strain, but a 150% increase in shear. The computational analysis opens up for improvements in models of shear-induced platelet activation, by offering an algorithm to distinguish shear from other modalities in intraventricular blood flow.

## 1 Introduction

Heart attack and stroke follow from a loss of blood supply to the heart and brain, respectively, often caused by a blood clot that obstructs a critical blood vessel, referred to as thrombosis. Blood clots are naturally formed as a response to injury to avoid blood loss, but may also form under other circumstances where the platelets (thrombocytes) of the blood are activated which leads to an increased tendency of the platelets to aggregate, see e.g., Previtali et al. (2011). Coronary thrombosis usually forms as a result of atherosclerosis in the coronary arteries, but platelet activation can also be triggered by contact with foreign material, such as a medical device, see e.g., Rojano et al. (2019), or by the mechanical stresses in the blood flow. Specifically, it is associated with elevated levels of shear in the blood. The degree and time extent of the activation is a function of the type of mechanical stimulus experienced by the platelets, see e.g., Ding et al. (2015); Rahman et al. (2020). Therefore, a detailed analysis of the structure of the blood flow may help to assess the risk for platelet activation and thrombosis. The heart is supplied blood through the right and left coronary arteries, with inlets through the right and left coronary sinuses which are part of the aortic root attached to the left ventricle (LV) of the heart. Further downstream the aorta, the brain is supplied blood through the carotid arteries that emanate from the aortic arch. As a consequence, the level of platelet activation in the LV blood flow is of interest when assessing the risk for thrombosis events in both the heart and the brain.

Blood flow in the LV is complex, in particular in diastole when the heart relaxes and blood flows into the ventricle from the left atrium through the open mitral valve. The ventricular blood flow is also highly sensitive to the structure and function of the mitral valve, and pathologies such as mitral valve regurgitation or stenosis, or treatments for these, can drastically change the flow pattern. In the early filling phase of diastole (the E-wave) a jet is formed which then breaks down in the diastasis phase, after which a second jet is formed in the atrial contraction phase (the A-wave). The two successive jets create shear layers and vortex rings in the ventricle, which then break down into turbulent flow structures on a range of scales. The blood flow generates mechanical stimuli on blood cells that trigger different responses in the cells, for example, activation of platelets. Mechanical stimuli on blood cells are difficult or impossible to assess by medical imaging alone, and the turbulent nature of the ventricular flow makes this challenge even greater. In this article we investigate to what degree a computational model can be used to assess the mechanical stimuli on blood cells in the LV flow, building on our previous work on patient-specific simulations of the LV blood flow, see e.g. Larsson et al. (2017b) and Larsson et al. (2017a), and analysis of turbulent flow structures, see Hoffman (2021). We introduce the triple decomposition of the velocity gradient tensor as a way to improve measures of intraventricular shear flow.

Attempts have been made before to evaluate shear levels and platelet activation in blood flow, often using the strain rate tensor in various models, see Han et al. (2022). Here we instead introduce a different tool to explicitly extract the shear: The triple decomposition of the velocity gradient tensor, which was first suggested by Kolář (2007) to improve the identification and visualization of vortices. Hoffman (2021) noted that the triple decomposition implies that the flow locally can be approximated by a sum of shear flow, rigid body rotational flow and irrotational straining flow. This decomposition has already proven useful in simpler model cases. Hayashi et al. (2021) simulated a temporally evolving turbulent planar jet, and applied the triple decomposition to it. Thus they were able to distinguish the shear from the rotation and straining flow to characterise small-scale shear layers. Nagata et al. (2020) used the triple decomposition to simulate fluid flow in a cube-shaped domain and demonstrated the distinction of the shear, rotation and straining flows. Building on these results which motivate the usefulness of the triple decomposition, we here take the next step by applying it in a bio-mechanical setting and showing how explicitly extracting the shear offers improvements to models of shear-induced platelet activation.

To demonstrate the usefulness of the triple decomposition we apply it in a context where treatment of a cardiac disease significantly changes the characteristics of the intraventricular blood flow, which could be expected to alter the levels of mechanical stimuli within the blood flow as well. The setting we choose for this is treatment of mitral valve regurgitation by mitral valve clip (MVC) intervention. Mitral regurgitation is a condition in which the mitral valve does not close properly and therefore may leak during systole when the valve is supposed to be sealed. A common procedure to mitigate mitral valve regurgitation is to suture the two leaflets of the mitral valve together using a MVC Mauri et al. (2013). Instead of a single inflow jet through the mitral valve (MV), the altered inflow profile creates two parallel inflow jets, which are more narrow than the original single jet. The changes in velocity due to smaller total inflow area, and shape of the inflow from one to two inflow jets, make up an interesting simulation case to apply the triple decomposition on.

Recently, patient-specific computer simulations have been used to evaluate the risks and benefits of a MVC intervention, see Caballero et al. (2020). Some clinical case studies have also suggested risks linked to blood shearing against the device itself, see e.g. Barrett et al. (2020). More generally, shear-induced platelet activation models have been used to estimate risks for thrombosis events in several settings, and these models are often based on a symmetric strain rate tensor, see Han et al. (2022), which fails to perfectly distinguish shear flow from straining flow. By using the triple decomposition instead, we here show that these flow modalities can be distinguished, and shear-induced platelet activation modelling may be improved. Similarly, the triple decomposition has previously been demonstrated to provide an improvement to vorticity-based vortex identification methods by distinguishing rotation from shear, see Liu et al. (2019).

In this article, we use a finite element method to simulate the blood flow in the LV before and after a MVC intervention, to analyze the difference in the ventricular blood flow. We also perform a sensitivity study with respect to the positioning of the MVC. A two-dimensional model of the mitral valve is used, which represents an approximation of the projected opening between the left atrium and the left ventricle. The mitral valve model is then modified to take the positioning of the MVC into account. Note that our aim is not to make any claims about any potential risks connected to MVC intervention. The purpose of simulating MVC treatment in this setting is to provide a simulation case which alters the structure of the inflow of blood into the LV, to illustrate how such structure changes are captured by the triple decomposition.

In Section 2 we describe the computational model and the triple decomposition of the velocity gradient tensor, and in Section 3 we present results from the simulations. These results are then discussed in Section 4.

## 2 Method

### 2.1 Left Ventricle Model

The LV model is based on 4D transthoracic echocardiography (TTE) images of the left ventricle of one human subject, acquired using the technique described in detail in Larsson et al. (2017b). Images were captured in 26 frames over a full cardiac cycle which lasted for 830 ms, and the heart wall was segmented in a semi-automated fashion in each image, see Hansegård et al. (2009). From this a set of triangulated surface meshes were generated which described the deformation of the endocardium over the cardiac cycle.

The orifice of the mitral valve (MV) was identified by a sonographer in thirteen 2D short axis B-mode views in early diastole, with each view rotated 15° around the LV long axis. Similarly, the aortic valve (AV) opening was identified in a single 3-chamber B-mode view in early systole. Both valve openings were marked on the surface meshes.

From one surface mesh corresponding to end diastole, here referred to as the initial surface mesh, a refined version was produced, from which a tetrahedral volume mesh of the interior of the ventricle was generated. This volume mesh consists of 406,964 vertices. The maximum cell diameter in this mesh at end diastole, i.e. when the ventricle is at its maximum volume, is 1.45 mm. The minimum cell diameter at end systole is 0.56 mm, and the average cell diameter throughout the heartbeat cycle is 0.72 mm. This resolution was chosen based on the convergence studies in Larsson et al. (2017b), which showed less than 2% deviation in results when increasing mesh resolution above 400,000 vertices.

One of the 26 surface meshes generated from the TTE data diverged significantly from the others, and was thus considered an outlier and excluded from the simulations. Between the rest of the captured frames, the surface meshes were temporally interpolated using cubic Hermite interpolation, to yield one surface mesh for each simulated time step.

### 2.2 Simulation of Blood Flow

The blood in the LV is modelled as an incompressible Newtonian fluid. Although blood is known to express non-Newtonian properties in smaller blood vessels, see e.g., Pedley and Fung (1980), and Adjoua and Lucor (2019), the blood inside the heart chambers is typically modelled as a Newtonian fluid, see Westerhof et al. (2010). To simulate the LV blood flow, we determine the velocity vector **u**(**x**, *t*): Ω^*t*^ → ℝ^3^ and scalar pressure *p*(**x**, *t*): Ω^*t*^ → ℝ, such that the Navier-Stokes equations are satisfied:

$$\rho \left(\overset{\cdot }{u}+\left(u\cdot \nabla \right)u\right)-\mu \Delta \mathrm{u+}\nabla p=0,\;\;\left(\mathrm{x,}t\right)\in \Omega^{t}\times \left[0,T\right]$$ (1)

$$\nabla \cdot \mathrm{u=}0,\;\;\;\;\;\;\;\;\;\;\left(\mathrm{x,}t\right)\in \Omega^{t}\times \left[0,T\right],$$ (2)

defined over the time-dependent domain Ω^*t*^ ⊂ ℝ^3^ at time *t* ∈ [0, *T*]. Eq. 1 expresses conservation of momentum, and Eq. 2 conservation of mass. Standard mathematical notation is used, with ∇ the gradient operator and ∆ the Laplacian operator. We use the dynamic viscosity *µ* = 0.0027 Pa·s and the blood density *ρ* = 1,060 kg/m^3^, taken from Di Martino et al. (2001).

Ω*^t^* represents the deforming LV, with the boundary ∂Ω^*t*^ partitioned into the heart wall and the two valve orifices. We use no-slip velocity boundary conditions, corresponding to setting the blood flow velocity equal to the deformation velocity of the heart wall and closed valves. In systole, the open AV is modelled by a standard outflow boundary condition, where the pressure is set to zero. It is modelled as fully open throughout systole, and completely closed during diastole. The model of the mitral valve is described below, with and without a MVC intervention. To simulate the LV blood flow over the cardiac cycle, the domain Ω^*t*^ is approximated by the tetrahedral volume mesh which is deformed over time based on the 25 surface meshes. For a detailed description of this process, see Larsson et al. (2017b). The Navier-Stokes equations are solved on this deforming volume mesh using an arbitrary Lagrangian-Eulerian (ALE) formulation of a stabilized finite element method, which is described in Hiromi Spühler and Hoffman (2021). Although the mesh deforms significantly throughout the heartbeat cycle, the usage of one Laplacian and one elastic mesh smoothing algorithm successfully eliminates the need for any remeshing during simulation. The elements are interpolated linearly, and throughout the simulations a time step of 1 ms is used. The Crank-Nicolson method is used for time integration.

### 2.3 Mitral Valve Model

We extend the LV model with a simple planar, data-driven parameterized model of the MV dynamics, which acts as a time dependent inflow boundary condition. The MV is modelled as a time-varying orifice representing the area enclosed by the planar projection of the leaflets onto the mitral annulus, accounting for virtual folding/unfolding mitral leaflets, similar to the work in Imanparast et al. (2016).

**FIGURE 1:**
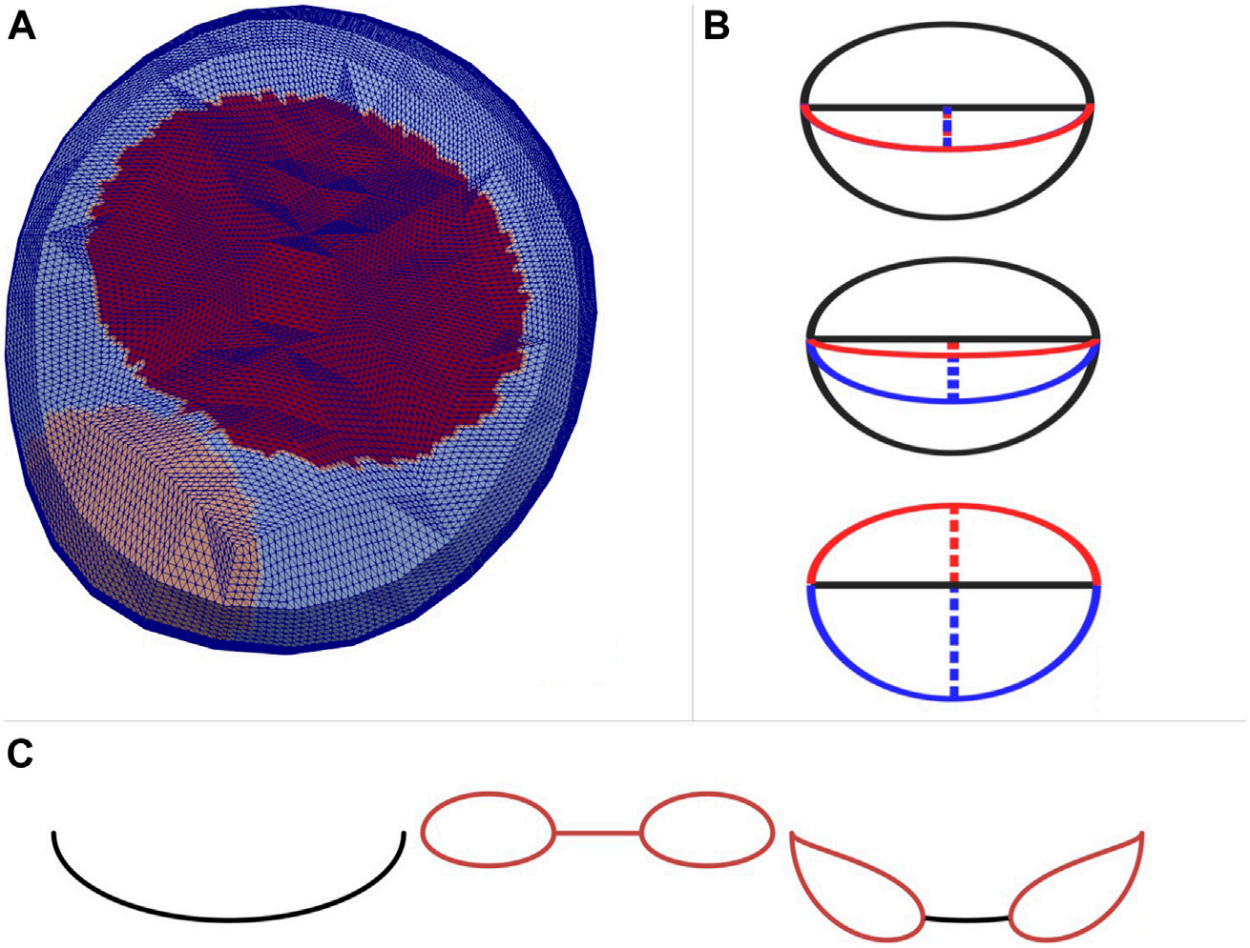
**(A)** AV placement (tan) and MV placement (red) on the LV surface mesh in an axial view. **(B)** The two half ellipses making up the MV model at different stages throughout the heartbeat cycle. The solid red line is the projection of the anterior leaflet, and the solid blue line the posterior. The dashed lines are the short axes of the corresponding half ellipse. In the top view the valve is fully closed (systole), and the leaflets overlap on the coaptation line. In the bottom view the valve is fully open (E-wave peak), and the center view shows early diastole. The solid black line through the valve model is the long axes of the two half ellipses. **(C)** The coaptation line (left), the two double half ellipses making up the MV model after MVC intervention (center) and the final shape of the MV model after MVC intervention (right). The part that does not open is the location of the clip.

The basic shape of the annulus is modelled as two half ellipses sharing a common long axis. From the ultrasound identification of the MV, the length of the long axis and the two short axes are identified, as well as the spatial 2D location of the coaptation line within the annulus area. An MV center point on the wall of the ventricle mesh is also given from the TTE images, which is used as a spatial reference point on the mesh to keep the MV in place. In Figure 1A the MV and AV areas on the simulation mesh are displayed, as well as the contour of the MV model and the projected leaflets for a particular moment in time in Figure 1B.

**FIGURE 2:**
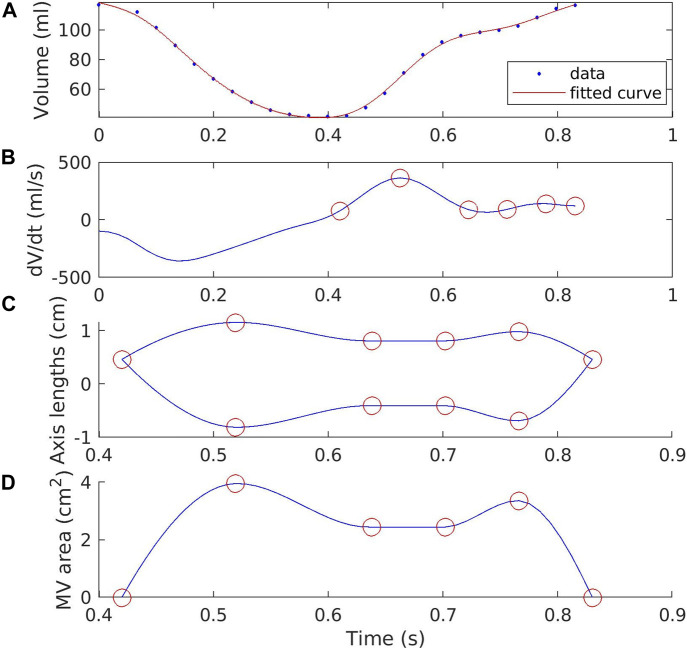
Volume of the acquired surface meshes of the LV, and the derivative of the interpolated curve. **(A)** shows the volumes of the 25 used frames directly from the TEE images as blue dots, and a cubic interpolation of these as a red line. **(B)** shows the first derivative, and the resulting timings from left to right of diastole start, E-wave peak, diastasis start and end, A-wave peak, and diastole end as red circles (see Table 1 for exact values). **(C)** shows the lengths of the short axes of the leaflets in the MV model over time, with the red circles marking the same instants as in **(B)**. **(D)** shows the resulting MV opening area in diastole for the untreated MV, again with red circles denoting the same timings. Note that the time scale in **(C)** and **(D)** only covers diastole, since the MV is closed in systole. The exact times and scalings of the short axes are given in Table 1.

The opening and closing of the MV are given as temporal variations of the short axes of the two half ellipses. During systole, the short axes are equal, effectively keeping the projection of the leaflet edges overlapped along the coaptation line. Throughout diastole, the short axes are scaled to imitate a projection of the moving leaflets onto the surface mesh. Effectively this corresponds to a maximum opening area at E-wave peak, slightly smaller in diastasis, and somewhere in between at A-wave peak. For these points in time in the diastole timeline, the short axis scalings are determined by an idealized MV, see Fyrenius et al. (2001). Patient-specific scalings are difficult to determine from echocardiography images since the resolution is limited. The predetermined scale factors are given in Table 1 for E-wave peak, diastasis start and end, and A-wave peak. The temporal variation of the axis lengths are also displayed in Figure 2C, and the resulting MV area in Figure 2D.

**TABLE 1:** Timings of different parts of diastole, and scalings of the short axis anterior (SAA) and short axis posterior (SAP) of the MV model at those times. The timings are determined by analyzing changes in the rate of expansion of the LV. Note that the coaptation line does not coincide with the intercommissural line (long axes), but the scalings at diastole start and end correspond to both leaflet edges coinciding with the coaptation line, i.e. the MV is closed at these times.

	Diastole Start	E-wave Peak	Diastasis Start	Diastasis End	A-Wave Peak	Diastole End
t (s)	0.420	0.525	0.644	0.711	0.779	0.830
SAA scale	0.40	1.00	0.50	0.50	0.85	0.40
SAP scale	-0.57	1.00	0.70	0.70	0.85	-0.57

Although the patient-specific scaling of the short axes could not be determined from the echocardiography images, the timings for the E-wave and A-wave peaks, as well as diastasis start and end, are determined based on the expansion rate of the LV of the particular subject studied here. This can be done patient specifically for other cases as well.

The volumes of the 25 frames extracted from the echocardiography images are displayed in Figure 2A, plotted against time from the beginning of systole. A cubic smoothing spline curve, *V*(*t*) is fitted to the data points. From the derivative *dV/dt* of this smooth curve, seen in Figure 2B, the timings are identified. Fundamentally, the shift from systole to diastole is identified as the point where *dV/dt* = 0, i.e. the rate of the volume change shifts from negative (contraction) to positive (expansion). The switch from diastole to systole is given simply as the beginning/end time of this heartbeat. Note that the isovolumetric phases between diastole and systole are excluded for simplicity.

The E-wave and A-wave peaks are easily identified as the local maxima in *dV/dt*, with E-wave peak being the earlier and higher one. To define diastasis, the local minimum between these two peaks is determined. Diastasis is then defined as the time between the two original data frames from the TTE images on either side of this local minimum. These timings are listed in Table 1.

With the timings of these four crucial points determined, along with the timings for shifts from diastole to systole and vice versa, the scaling of the short axes of the MV model are given by cubic Hermite interpolation between the scaled axis lengths and times given in Table 1. The resulting axes scaling over the full duration of diastole is displayed in Figure 2C.

#### 2.3.1 Mitral Valve Clip Intervention

To simulate the inflow of blood after a MVC intervention, part of the coaptation line of the MV was kept stationary in its closed state throughout diastole. This produces a valve model with two smaller openings, one on either side of the clip as displayed in Figure 1C. This is achieved by creating two smaller openings consisting of two half ellipses each, similar to the untreated case, and superpositioning this with the coaptation line. The two smaller openings behave much like smaller versions of the untreated MV model throughout diastole. They follow the same pattern for opening and closing, i.e. the same scalings and timings given in Table 1. In its closed state, the clipped MV model has the same coaptation line as the untreated valve model.

Since the length of the coaptation line is the same for the clipped and untreated models, the long axes of the two smaller openings are directly given from the remaining unclipped parts. Additionally, the relationship between the anterior and posterior short axis of each opening is the same as the relationship of the short axes in the untreated case. The length of the two anterior short axes are assumed to have the same relationship as the length of their corresponding long axes, and the same holds for the posterior short axes.

To compute the maximum length of the short axes in the MVC case, the length of the edge of the openings at E-wave peak is assumed to be the same as the length of the edges in the untreated case, i.e. the circumference of the orifice. Along with the relationships between different axes described above, this is used to uniquely determine the maximum length of the short axes. As in the untreated case, this is assumed to imitate a projection of the 3D MV opening onto the surface mesh of the ventricle, see Imanparast et al. (2016). Given the lengths of the ellipse axes and the MVC, the total area of the two openings were computed and are presented in Table 2.

**TABLE 2:** Position scale constants for the MVC simulations and resulting MV opening areas at E-wave peak. The MVC is positioned at *s*·*l* from the center of the coaptation line, with *l* being the length of the long axis in the MV model. Here *s* < 0 corresponds to clip positions anterolaterally (i.e. toward the right edge in Figure 1A) of the center, and *s* > 0 posteromedially (to the left in Figure 1A). For reference, the area of the MV opening at mid diastole with no MVC is 2.54 cm^2^.

*s*	-0.4	-0.2	0.0	0.2	0.4
Area (cm^2^)	1.08	0.93	0.86	0.93	1.08

### 2.4 Simulations

Five different cases were simulated, with MVC centers positioned along the coaptation line at *s*·*l* from the coaptation line center, with *l* being the same long axis length as used in the untreated valve, i.e. half the intercommissural diameter, and *s* ∈ (−1, 1) a scaling parameter. The values of *s* used in these simulations are given in Table 2, along with the resulting MV opening areas at mid diastole. Hence, *s* = 0 corresponds to the clip being in the center of the valve and *s* = ±1 to it being on the posteromedial/anterolateral commissure of the valve respectively.

In addition to the five cases with the MVC in different positions, one case with an untreated valve was also simulated using the original MV model. Note that the untreated valve was modelled without any regurgitation. This is a model simplification motivated by the fact that our focus is to compare the blood flow into the ventricle during diastole. In this model the inflow is not greatly affected by any regurgitation in systole. The significant difference is that between the flow in the different double orifice cases in MVC treated valves and the single orifice of the untreated valve.

The LV used in these simulations has a volume at end diastole of 117 ml, and an ejection fraction of 64%. These values, as well as the deformation of the ventricle volume throughout the heartbeats, are the same in all simulations. The untreated MV has a long axis of 12.8 mm, and short axes of 11.6 mm (posterior) and 8.1 mm (anterior) respectively. This results in an opening area of 4.0 cm^2^ at E-wave peak, and 2.54 cm^2^ in mid diastole. This is a relatively small opening area, so the resulting velocities and pressures in the MVC simulations are likely higher than a typical in-vivo case. This could be the case for the magnitudes of rotation, shear and strain as well. In an ongoing clinical trial of MVC treatment, patients with MV area ≤ 4.0 cm^2^ in mid diastole were excluded ClinicalTrials.gov (2021). Normal healthy MV’s typically have opening areas ranging from 6.5 to 4.0 cm^2^ in mid diastole.

All simulations were run on the KTH supercomputer Beskow (2021), a Cray XC40 system with 67,456 cores distributed over 4,120 Intel Xeon E5-269 × 2.1/2.3 GHz CPUs. The simulations were run for six heartbeats, starting from early systole, all with identical wall motion from the captured TTE images. The first five heartbeats were run to cancel out effects from the initial conditions, and only data from the sixth heartbeat was analyzed and presented here.

All simulations were done with FEniCS-HPC HeartSolver. MATLAB was used to compute the MV timings and opening areas, for computing the Schur decomposition of the velocity gradients, and for data analysis. Paraview was used for visualisation and data analysis.

### 2.5 Triple Decomposition of Velocity Gradient Tensor

To analyze the blood flow in the ventricle we can visualize the flow velocity and pressure computed from the Navier-Stokes equations. It is also useful to analyze the velocity gradient ∇**u**, a tensor which describes the local spatial change in velocity at each time and position, defined by its components

$${\left(\nabla u\right)}_{ij}=\frac{\partial u_{i}}{\partial x_{j}}.$$ (3)

Near each point **x**_0_ ∈ Ω^*t*^, the velocity gradient can be used to construct a linear approximation

$$u\left(x\right)\approx u\left(x_{0}\right)+\nabla u\left(x_{0}\right)\left(x-x_{0}\right),$$ (4)

and the mechanical stimuli on cells can be derived from the velocity gradient.

A standard double decomposition of the velocity gradient constitutes a separation of the flow into a straining flow and a rotational flow, corresponding to its symmetric part *S*(**u**), the strain rate tensor, and the skew-symmetric part Ω(**u**), the spin tensor:

$$\nabla \mathrm{u=}\frac{1}{2}\left(\nabla \mathrm{u+}{\left(\nabla u\right)}^{T}\right)+\frac{1}{2}\left(\nabla \mathrm{u-}{\left(\nabla u\right)}^{T}\right)=S\left(u\right)+\Omega \left(u\right),$$ (5)

where the superscript *T* represents the transpose of the tensor. The weakness of the double decomposition is that shear flow is unaccounted for. To address this shortcoming, the triple decomposition was proposed by Kolář (2007), where the velocity gradient tensor is decomposed into a sum of three parts corresponding to irrotational straining flow (i.e. compression and elongation) (∇**u**)_*EL*_, rigid body rotational flow (∇**u**)_*RR*_, and shear flow (∇**u**)_*SH*_:

$$\nabla \mathrm{u=}Q\bar{\nabla u}Q^{T}=Q\left({\left(\nabla u\right)}_{EL}+{\left(\nabla u\right)}_{RR}+{\left(\nabla u\right)}_{SH}\right)Q^{T},$$ (6)

where *Q* is an orthogonal matrix that represents a new frame of reference in which the shear flow can be subtracted from the other two parts. For the sake of brevity, we will refer to the three flow structures as strain, rotation and shear.

Algebraically, the matrix decomposition may be interpreted as a sum of a normal symmetric part, a normal skew-symmetric part, and a non-normal part, which can be computed through the real Schur decomposition of the velocity gradient tensor, see e.g., Van Loan and Golub (1996). The real Schur decomposition yields the orthogonal matrix *Q*, and an upper quasitriangular matrix $\bar{\nabla u}$ . To decompose $\bar{\nabla u}$ into the components in Eq. 6 is simply a matter of subtracting the symmetric and skew-symmetric parts from the non-normal part

$$\bar{\nabla u}=\left[\begin{array}{ccc} \lambda & \in & \zeta \\ 0 & \alpha & \beta \\ 0 & \gamma & \alpha \end{array}\right]=\left[\begin{array}{ccc} \lambda & 0 & 0\\ 0 & \alpha & 0\\ 0 & 0 & \alpha \end{array}\right]+\left[\begin{array}{ccc} 0 & 0 & 0\\ 0 & 0 & -\gamma \\ 0 & \gamma & 0 \end{array}\right]+\left[\begin{array}{ccc} 0 & \in & \zeta \\ 0 & 0 & \beta +\gamma \\ 0 & 0 & 0 \end{array}\right]$$ (7)

Here *λ* is a real eigenvalue of ∇**u**, and the other two eigenvalues are given by the block

$$\left[\begin{array}{cc} \alpha & \beta \\ \gamma & \alpha \end{array}\right].$$ (8)

α is the real part of the complex eigenvalues, ± *γ* is the imaginary part, and |*β*| > |*γ*|. (Note that this is only true if there is rotation present in the mesh cell. If not, *γ* = 0 and the diagonal elements are not necessarily equal.) ∈, *ζ* and *β* + *γ* make up the non-normal part, which is maximized when subtracting the normal parts. This effectively means subtracting the pure strain and rotation from all of the shear, to not get any remaining shear contamination in the other parts.

In this article, the triple decomposition was computed by using the Matlab function *schur*. For further details on the computation of the triple decomposition, see Hoffman (2021) or Kronborg and Hoffman (2022). To determine the magnitude of each flow component, Frobenius norms of each respective matrix were computed.

### 2.6 Von Mises-like Scalar Shear Stress

Models of shear-induced platelet activation are typically based on so-called scalar shear stress (SSS), as described by Han et al. (2022). This estimate is based on the shear rate as defined from the velocity gradient by

$$\overset{\cdot }{\gamma }{\;}_{ij}={\left(\nabla u\right)}_{ij}+{\left(\nabla u\right)}_{ji}.$$ (9)

Han et al. (2022) then define the shear stress tensor as *τ_ij_* = *µγ˙ _ij_*, with *µ* the dynamic viscosity. However, to keep units consistent with our estimates of mechanical stimuli from the triple decomposition, we simply use

$$\tau_{ij}=\overset{\cdot }{\gamma }{\;}_{ij}.$$ (10)

The von Mises-like SSS is then defined by

$$\tau ={\left[\frac{1}{6}\left({\left(\tau_{xx}-\tau_{yy}\right)}^{2}+{\left(\tau_{yy}-\tau_{zz}\right)}^{2}+{\left(\tau_{xx}-\tau_{zz}\right)}^{2}\right)+\tau_{xy}^{2}+\tau_{yz}^{2}+\tau_{xz}^{2}\right]}^{1/2}.$$ (11)

This measure is thus based on the strain rate tensor *S*(**u**), defined as the symmetric part of the double decomposition in Eq. 5. The relation to the deviatoric strain energy is noted by Faghih and Sharp (2016), but the relation between strain and shear in this measure and its impacts on shear-induced platelet models are not expanded on by Han et al. (2022). As we shall see, the SSS contains components of both.

## 3 Results

### 3.1 Flow Structures

**FIGURE 3:**
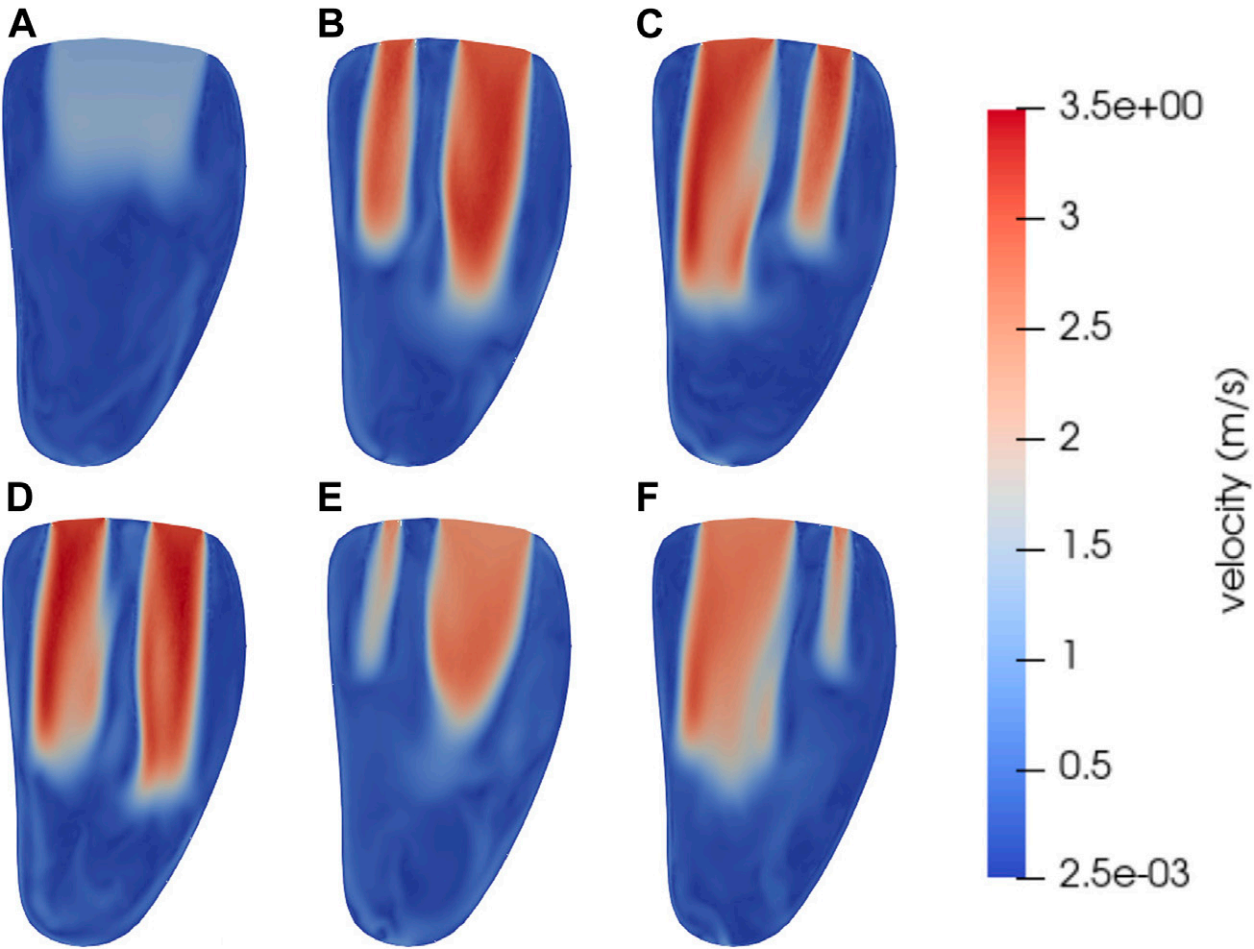
Snapshots of the velocity magnitude at time *t* = 0.45 s in a vertical bicommissural cut through the left ventricle. This is in early diastole before the E-wave peak, when the intraventricular blood flow is dominated by strong inflow jets. Subfigure **(A)** shows the untreated case. The other subfigures have MVC’s positioned at *s·l* from the center, with *l* being the long axis of the MV model. The scale factor *s* for the different cases are **(B)** *s* = −0.2, **(C)** *s* = 0.2, **(D)** *s* = 0.0, **(E)** *s* = −0.4, **(F)** *s* = 0.4. The corresponding MV areas for each case in mid diastole are given in Table 2.

Snapshots of the velocity magnitudes in a bicommissural vertical cut through the ventricle for all simulated cases are shown in Figure 3. The snapshots are taken at time *t* = 0.45 s from the start of the analyzed heartbeat (cf. Figure 2C), i.e. 30 ms after the start of diastole. At this point in time inflow jets through the mitral valve can be observed in all cases. The untreated valve has a single wide jet, whereas the cases with MVC’s in different positions show the double jets characteristic of inflow after MVC treatment.

The average velocity in the jet of the untreated case (Figure 3A), which has a larger MV opening area than the others, is smaller than the velocities in the clipped cases. This single wide jet also does not reach as far down into the ventricle as the jets in the other cases. Comparing the treated cases, the one with the clip in the center of the coaptation line (Figure 3D) has a slightly higher peak velocity than the others. This case is the one with the smallest total opening area, as given in Table 2. In the ventricles with asymmetrically placed clips, the wider of the two jets typically reaches further into the ventricle than the smaller jet. In all cases, treated as well as untreated, the Reynolds number of the inflow jet is of the order 10^4^, or slightly lower, using the diameter of the inflow area as length scale.

**FIGURE 4:**
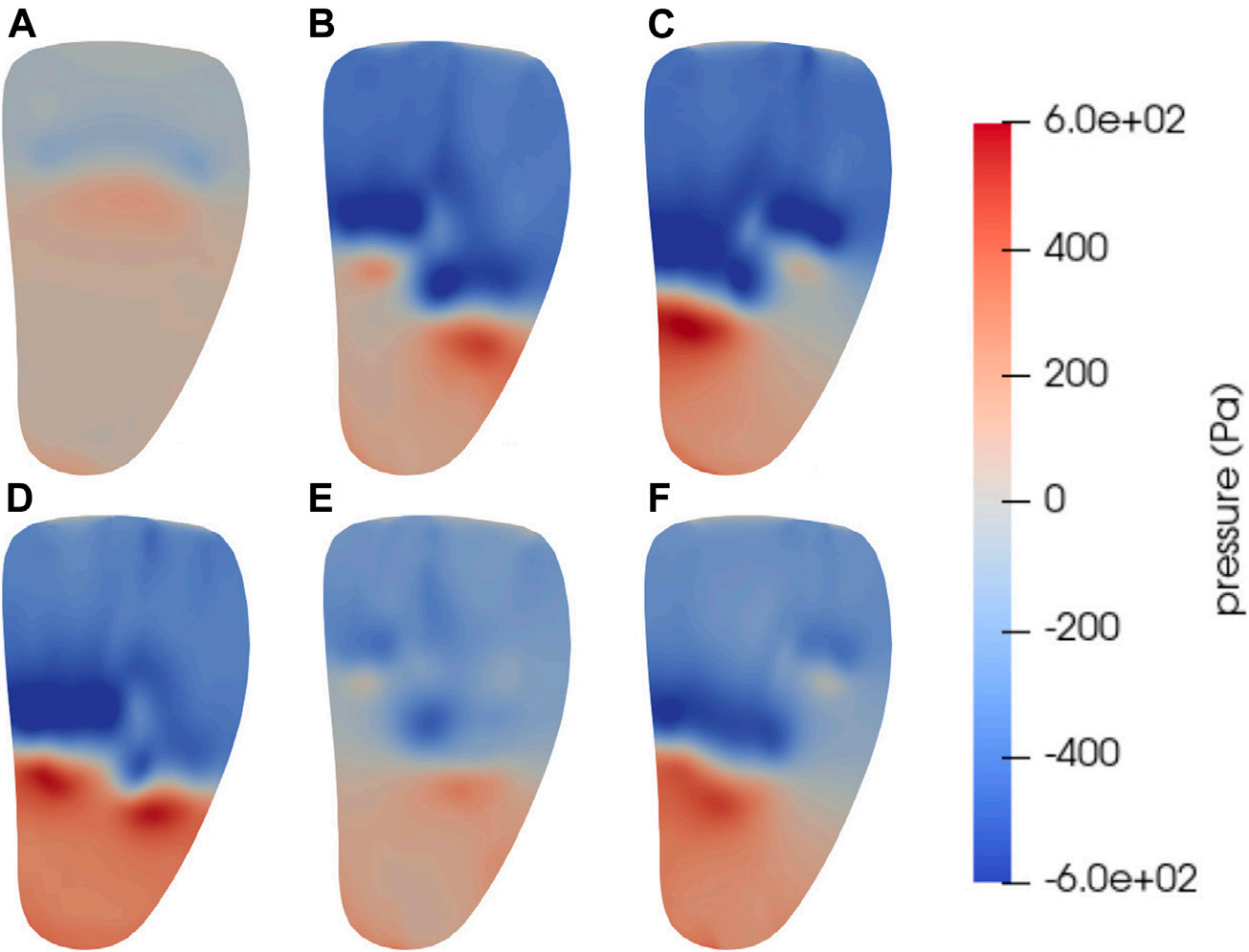
Snapshots of the pressure at time *t* = 0.45 s in a vertical bicommissural cut through the left ventricle. This is in early diastole before the E-wave peak, when the intraventricular blood flow is dominated by strong inflow jets. The regions just below the jets experience high pressure, while there is a pressure drop around the base of the jet. **(A)** shows the untreated case. The other subfigures have MVC’s positioned at *s·l* from the center, with *l* being the long axis of the MV model. The scale factor *s* for the different cases are **(B)** *s* = −0.2, **(C)** *s* = 0.2, **(D)** *s* = 0.0, **(E)** *s* = −0.4, **(F)** *s* = 0.4. The corresponding MV areas for each case in mid diastole are given in Table 2.

Snapshots of the pressure at the same instant and the same cut as the velocity described above are shown in Figure 4, with subfigure Figure 4A showing the untreated case and Figures 4B–F the MVC cases with clips positions given by Table 2. A recurring pattern of high pressure in the apical and mid-cavity parts of the ventricle is apparent in all simulated cases, with slight variations. In the untreated case (Figure 4A), where the jet does not extend as far into the ventricle, the overall pressure variations are lower than in the cases with MVC’s. In the MVC cases, the double jets extend to different depths in the ventricle, which can also be seen in the velocity in Figures 3B–F. This makes the line between high and low pressure less straight in the MVC cases, especially when the MVC is asymmetrically positioned. High pressures just below the jets are apparent, but the lowest pressures are found around the front of the jets. This indicates the presence of vortex rings in these parts, since pressure is typically low in vortex centers.

### 3.2 Triple Decomposition of the Velocity Gradient Tensor

Placing a clip in the MV significantly reduces its opening area, as can be seen in Table 3. A natural result of this is higher inflow velocities, which the table also displays, with higher velocities the smaller the opening is. Both the peak velocity and the peak pressure are mainly affected by the reduction in area, but not so much by which side the clip is placed on. This is however not necessarily the case for the triple decomposition components, as is evident from the same table.

**TABLE 3:** Peak values in the untreated simulation case, and the corresponding values for each of the MVC cases. Area is given at mid diastole. Velocity and pressure are the maximum values reached anywhere in the LV at E-wave peak. Rotation, shear and strain are the maxima reached in Figure 5.

Simulation Case	Untreated	-0.4	-0.2	0.0	0.2	0.4
Area at mid diastole (cm^2^)	2.54	1.08	0.93	0.86	0.93	1.08
E-wave peak velocity (m/s)	1.86	2.84	3.53	3.76	3.51	2.84
E-wave peak pressure (kPa)	0.667	2.396	3.631	4.640	3.621	2.993
Peak average rotation (s^−1^)	21.0	35.4	37.0	39.8	39.9	34.1
Peak average shear (s^−1^)	174.6	344.8	397.5	439.1	409.7	368.7
Peak average strain (s^−1^)	51.4	82.5	86.7	95.3	93.6	81.3

**FIGURE 5:**
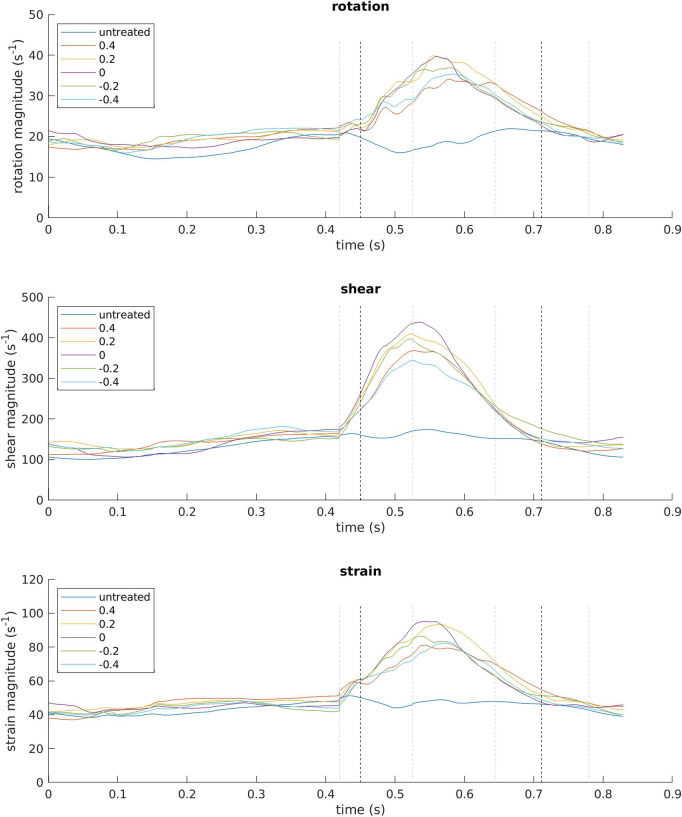
Magnitudes of rotation, shear and strain averaged over the entire ventricle volume and plotted against time over one heartbeat, starting with systole, for all simulated cases. The legend indicates the location of the MVC as relative shift from the center of the coaptation line, except for the healthy (untreated) case. The gray dashed lines mark the time of the start of diastole, E-wave peak, diastasis start, and A-wave peak, also given in Table 1. The black dashed lines mark *t* = 0.45 s and *t* = 0.71 s, i.e. the times at which the blood flow is visualized in Figures 3, 4, 6–9.

Peak velocities and pressures in Table 3 are the highest values achieved in any point in the ventricle at the time of the E-wave peak, i.e. *t* = 0.53 s, when the expansion rate of the ventricle is at its peak. At this point in time the blood flow in the LV is dominated by the inflow jet from the atrium. The values for rotation, shear and strain in the same table are computed by instantaneous spatial integration of each component over the LV volume, and normalizing by dividing with the volume to remove any effects of differing ventricle size at different points in time, thus achieving a time-dependent spatial average (also visualized in Figure 5). This spatial averaging diminishes the effects of any extreme spatial maximum values. The values presented in Table 3 are the maxima of these averages reached at any point throughout the heartbeat cycle. With the exception of the rotation in the untreated case, which peaks during the A-wave, all these maxima occur during the E-wave.

With the clip placed in the center of the MV, the opening area is the smallest out of all these simulation cases (see Table 3). For shear and strain, this leads to the highest computed peak values, but the peak rotation magnitude is actually slightly larger with the clip placed slightly posteromedially of the center. It is true for all three components that the peak values with the clip displaced to either side are not only dependent on how far the clip is displaced, but the direction as well. However, in most cases it does seem like smaller opening area does contribute to higher magnitudes of rotation, shear and strain, although the effect varies. The MV with the clip in the center has an opening area of 34% of that of the untreated MV. Its peak average rotation and strain are both roughly 190% of those for the untreated case, but its peak average shear is about 250% of that for the untreated case. As a reference, the maximum velocity increased about twofold in the same case. Throughout all simulation cases, shear has the highest relative increase when treated with MVC, whereas rotation and strain increase roughly equally.

**FIGURE 6:**
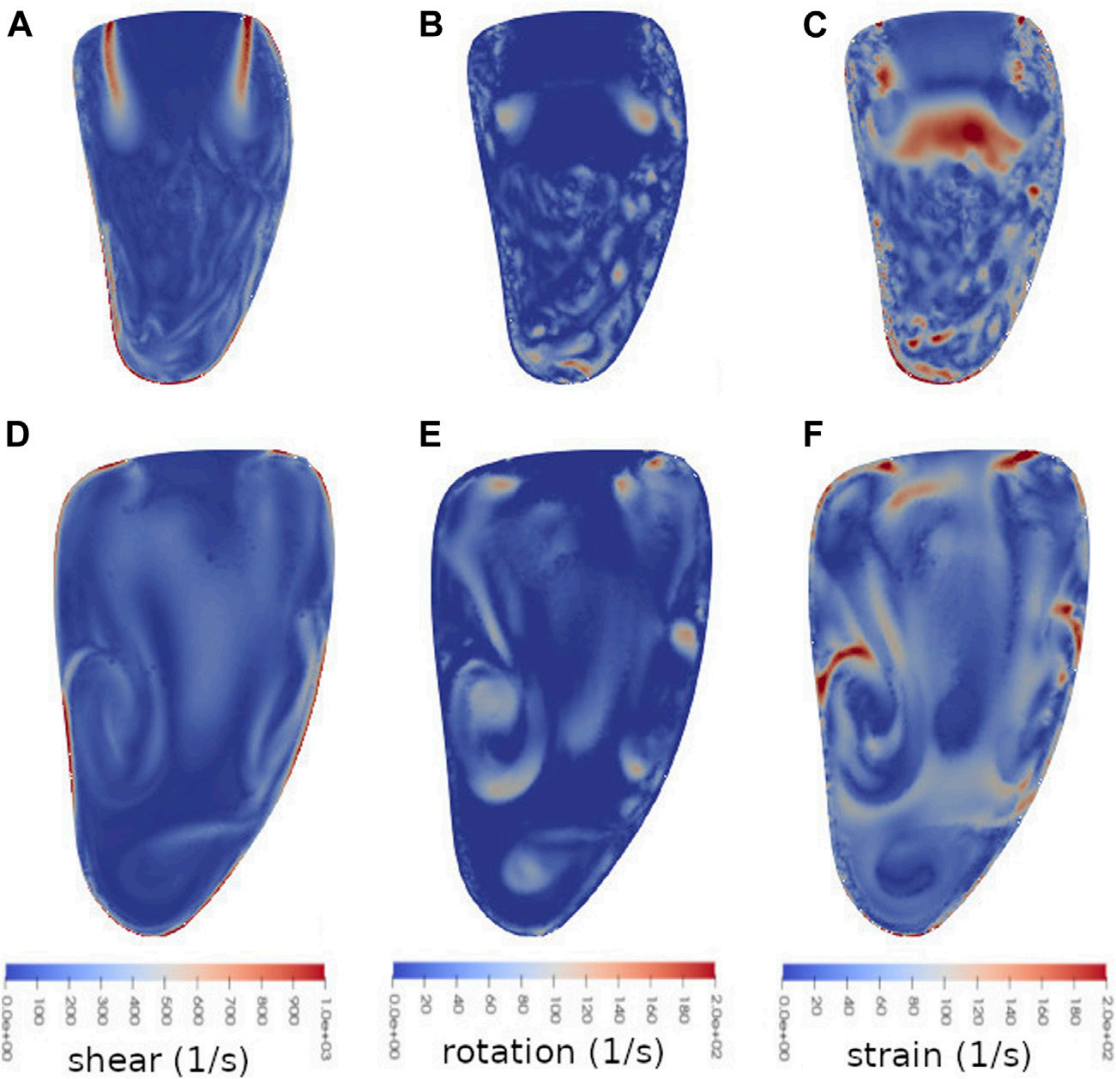
Snapshots of shear flow **(A** and **D)**, rotational flow **(B** and **E)** and straining flow **(C** and **F)** at two times in during diastole: *t* = 0.45 s **(A,B** and **C)** and *t* = 0.71 s **(D,E** and **F)** in a vertical bicommissural cut through the left ventricle. This simulation was done with an untreated valve. At time *t* = 0.45 s (early diastole), the flow is dominated by the incoming E-wave jet. The velocity and pressure at this time are shown in Figures 3A, 4A respectively. At time *t* = 0.71 s (end diastasis) the inflow jet is weaker, and the initial dominant flow structures of the E-wave have started to break down. Note that the different sizes of the ventricle are due to the diastolic expansion.

There are significant differences in how the flow in the ventricle behaves when dominated by strong inflow jets, i.e. during E-wave, and when there is no single dominating structure dictating most of the flow. Figure 6 shows snapshots of the three modalities of the triple decomposition, i.e. rotation, shear and strain, in the simulation case without the MVC. The components are displayed at two different times in diastole: *t* = 0.45 s, which corresponds to the velocities shown in Figure 3, and *t* = 0.71 s, which is the time of the end of diastasis. Thus the first snapshots (Figures 6A–C) show the triple decomposition at a point in time when the flow is dominated by the strong inflow through the MV in early diastole, and the second snapshots capture the components after the initial E-wave jet has broken down into smaller flow structures in the ventricle.

At *t* = 0.45 s, it is clear that different flow modalities are prevalent in different parts of the ventricle. Rotation (Figure 6B) appears in small connected areas in the lower part of the ventricle, but notably also on either side of the lower part of the jet structure, showing the presence of a vortex ring forming around the jet. Within the jet itself, where the flow is roughly unidirectional, the rotation is 0.

Shear (Figure 6A) at the same moment is high at the outer edge of the jet, and along the lower wall of the ventricle. There are weaker shear structures throughout much of the rest of the ventricle as well, but none within the jet. Strain (Figure 6C) is also large along the wall around the apical part of the ventricle. Additionally, below the jet, at the front of the blood flowing into the ventricle from the atrium, straining flow is high. There are also smaller spots throughout the ventricle with relatively high or low strain.

At *t* = 0.71 s, when the E-wave jet has diminished and there is no single strong feature dominating the flow field, the overall distribution of the different flow modalities is more mixed. Rotation (Figure 6E) is loosely connected in structures throughout the ventricle. Shear (Figure 6D) and strain (Figure 6F) are still relatively large along the ventricle wall around the apex, but the shear now also extends to other parts of the wall. In some structures near the wall but extending into the interior of the ventricle the strain is relatively high.

**FIGURE 7:**
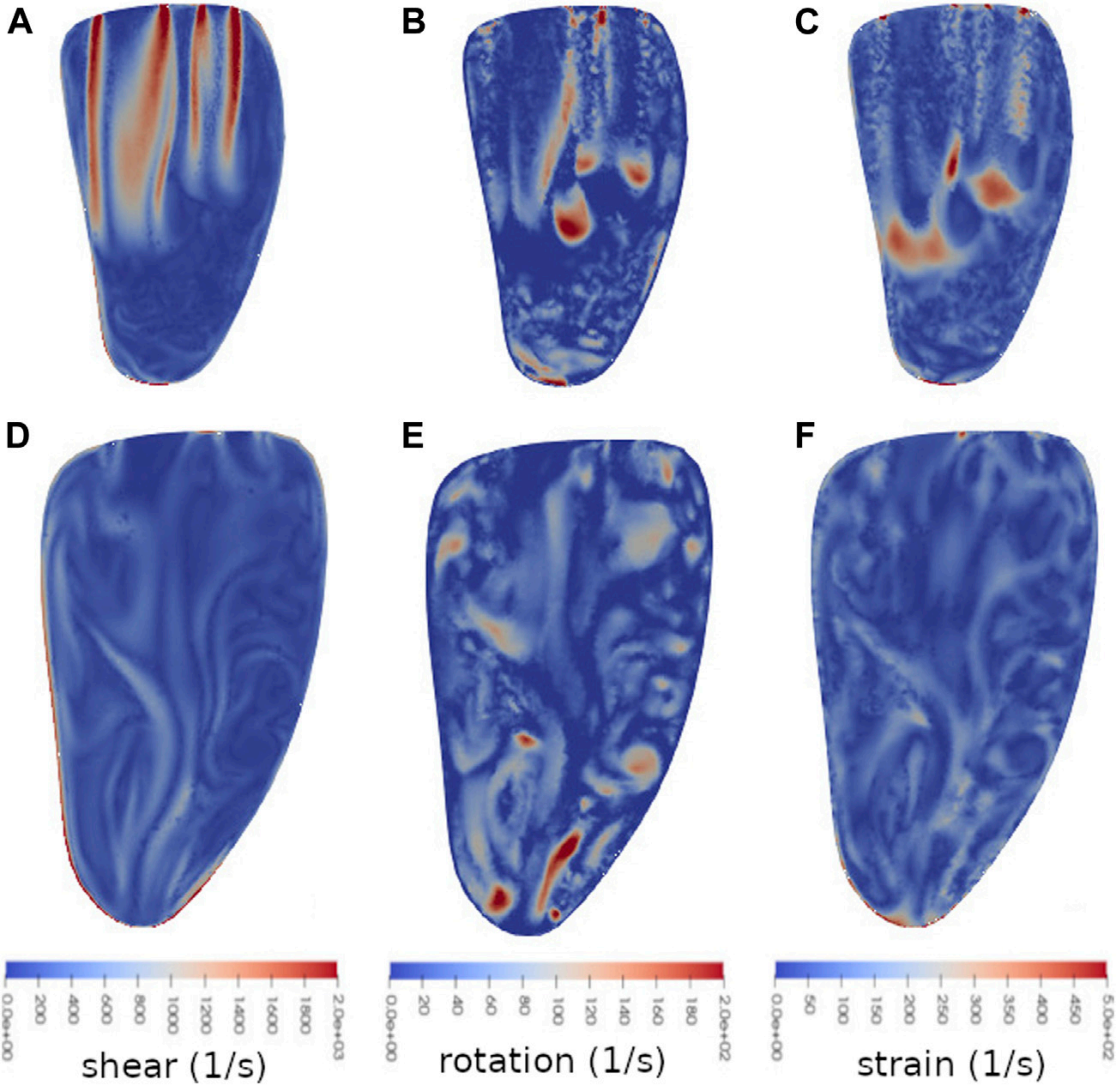
Snapshots of shear **(A** and **D)**, rotation **(B** and **E)** and strain **(C** and **F)** at two times during diastole: *t* = 0.45 s **(A,B** and **C)** and *t* = 0.71 s **(D,E** and **F)** in a vertical bicommissural cut through the left ventricle. This simulation was done with MVC placed at *s·l* from the center of the coaptation line, with *l* being the long axis in the MV model, and the position scale factor *s* = 0.2. At time *t* = 0.45 s (early diastole), the flow is dominated by the two incoming E-wave jets. The velocity and pressure at this time are shown in Figures 3C, 4C respectively. At time *t* = 0.71 s (end diastasis) the inflow jets are weaker, and the initial dominant flow structures of the E-wave have started to break down. Note that the different sizes of the ventricle are due to the diastolic expansion.

When it comes to the cases treated with MVC’s, some of the appearing flow structures share similarities with those in the untreated case. Snapshots of rotation, shear and strain for the MVC case with position scaling *s* = 0.2 (corresponding to case in Figures 3C, 4C) are displayed in Figure 7. The snapshots are taken at the same moments in time as the ones in Figure 6, and show the same cut through the ventricle. Note however the different scales for shear and strain.

With the flow profile dominated by two jets, as in early diastole in Figures 7A–C, the rotation (Figure 7B) again shows structures reminiscent of vortex rings around the lower part of the jets. (For the corresponding velocity snapshot, see Figure 3C.) However, the magnitude of the vortex rings appear larger for the parts in the interior of the ventricle than where the jet runs close to the wall.

Along the edges of the jets, high shear is visible in the clipped case as well (Figure 7A). The shear of the wider jet seems to extend into the center of the jet as well, but this is because the displayed cut is not perfectly through the center of the jet. Like in the untreated case, there is also significant shear along the wall in the lower part of the ventricle.

The strain in the clipped case (Figure 7C) is once again large at the bottom of the jet where the inflow meets slower moving blood. It is also high at the wall in the apical part of the ventricle, and at the edges of the MV openings. There is also an area of higher strain right in the center of the ventricle, between the two jets.

At the time of the second snapshot, i.e. the end of diastasis when the strongest effects of the initial E-wave jets have diminished, the rotation structures are fairly well distributed in the ventricle, with some areas in the apical part of the ventricle where the rotation is relatively high (Figure 7E). Much like in the untreated case, the shear (Figure 7D) extends along more parts of the ventricle wall in the second snapshot, with weaker effects in the interior part. The strain (Figure 7F) retains its high magnitude along the apical part of the ventricular wall.

Differences between the triple decomposition components in the treated and untreated cases include, perhaps most notably, significantly higher magnitudes in strain and shear in the clipped case. The magnitude scales here have been adjusted to better show the location and structures of the different modalities, but Figure 5 indicates that the claim holds. In the snapshots of the early diastolic flow, interactions between the two jets in the clipped case give rise to effects not seen in the untreated case, such as the high strain in the center of the ventricle. But vortex rings, high shear along the edge of the jets, and high strain at the bottom of the jets appear in both the treated and untreated cases. Shear and strain along the walls are also prevalent in both cases.

### 3.3 Comparison Between Shear Flow and von Mises-like SSS

**FIGURE 8:**
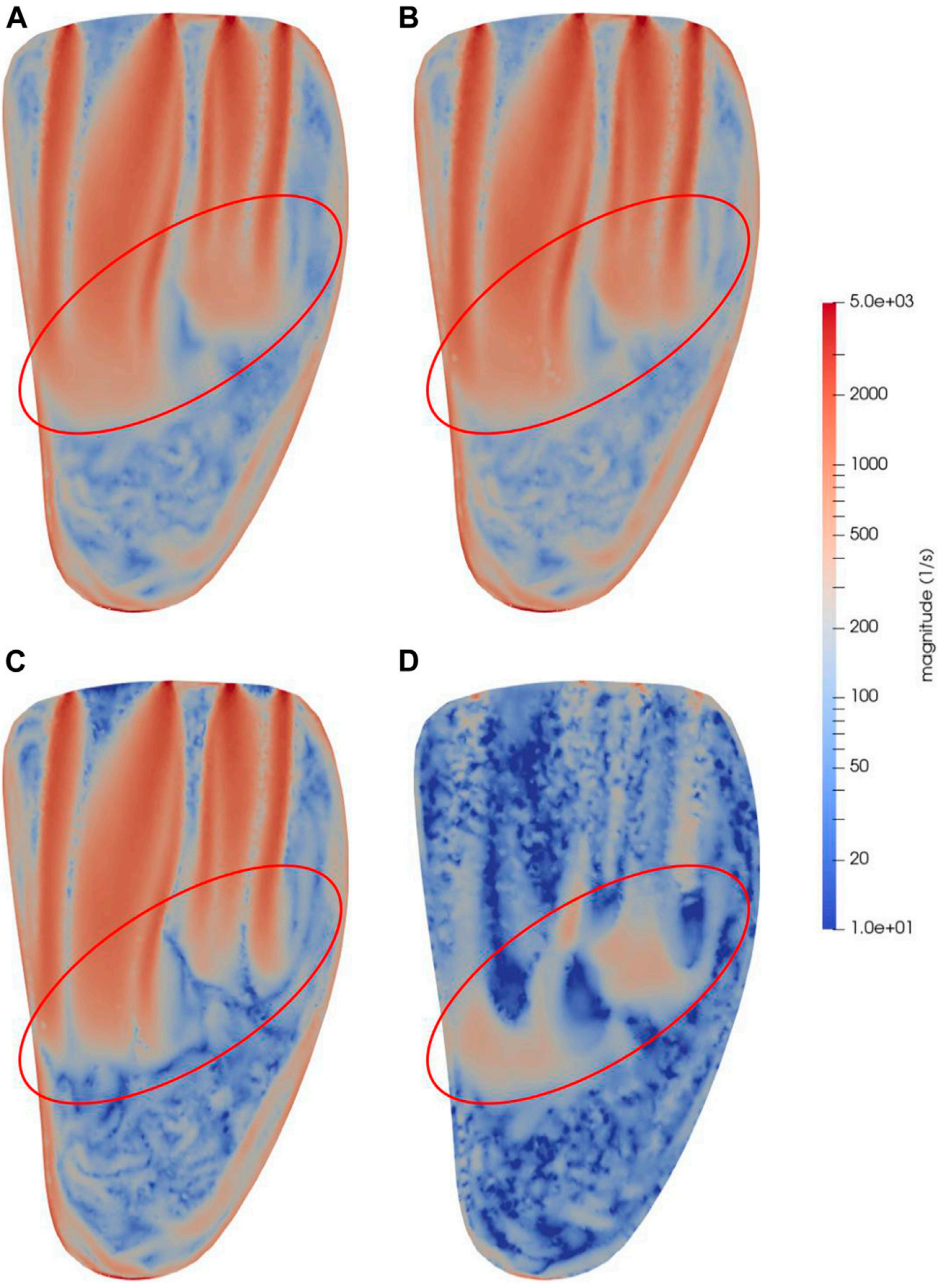
Snapshots of **(A)** von Mises-like SSS, **(B)** shear and strain summed, **(C)** shear and **(D)** strain at time *t* = 0.45 s in a bicommissural vertical cut through the left ventricle. This simulation was done with a MVC placed at *s·l* from the center of the coaptation line, with *l* being the long axis in the MV model, and the position scale factor *s* = 0.2. The red ellipses indicate significant areas of particularly high strain, to highlight the difference between SSS and shear computed using the triple decomposition. The velocity and pressure at this time are shown in Figures 3C, 4C respectively.

To tie the triple decomposition results back to earlier methods of platelet activation modelling, Figure 8 shows a snapshot of the SSS (Eq. 11) computed using the strain rate tensor as described by Han et al. (2022) and Faghih and Sharp (2016), alongside the shear and strain components computed using the triple decomposition. Figure 8B also shows the shear and strain components added together. A logarithmic scale is used to more clearly illustrate areas of significant strain in a scale that also encompasses the shear range. The SSS (Figure 8A), which is commonly used in models of shear-induced platelet activation and claimed to be a measure of the shear, is visibly more similar to the sum of the shear and strain components (Figure 8B) than to the shear alone. The circled parts indicate the front of the jet where the strain is large, but where the shear (Figure 8C) is actually much more localized than the SSS indicates. The shear alone does not reach as deep down towards the apical part of the ventricle as the SSS indicates. The strain however, which is predominantly large around the front of the jets, reaches slightly further down.

### 3.4 Temporal Variations of Flow Modalities

To get an idea of the duration and intensity of the different flow modalities from the triple decomposition over a full heartbeat, Figure 5 shows the magnitude of the rotation, shear and strain for all simulated cases over time. There are some similarities apparent between all modalities. In systole, they all vary much less than in diastole, with the exception of the untreated case. Across all modalities, the magnitudes in the untreated case peak at much lower values than the MVC cases, despite being at similar levels in systole. It is difficult to distinguish any clear differences between the simulation cases in systole, which is not surprising since all cases have the same AV opening and the same movement of the ventricle wall.

In diastole, all modalities follow similar temporal patterns in all MVC cases. With the increasing influx of blood in early diastole, the magnitudes of the rotation, shear and strain rise. Right at the start of diastole there is an initial jump in magnitude for all three modalities. This is the result of a few frames with very small MV opening areas, as the valve is just starting to open, resulting in relatively high inflow velocities even though the actual volume expansion is small. Shortly after the E-wave peak, the magnitudes also peak and then start to fall. They continue decreasing towards systolic levels, with no real noticeable effects from the A-wave. However, despite having the same inflow volume of blood as the MVC cases, the magnitudes of rotation, shear and strain do not rise high above the systolic levels in the untreated case. In fact, after a small initial rise right at the start of diastole, they drop during some of the time leading up to the E-wave peak.

## 4 DISCUSSION

### 4.1 Characteristics of Flow Modalities

The triple decomposition used here gives an excellent view of the locality and structure of different flow modalities in the LV blood flow. Structures such as strong inflow jets can be visualized and quantified by studying the velocity, and with the addition of pressure, phenomena like the vortex rings around jets can be visualized. However, studying e.g. the shear around the jets requires more sophisticated tools. The triple decomposition captures this, as well as confirms expectations from the velocity and pressure plots. The pressure in the untreated case is relatively low in the basal part of the ventricle, but slightly higher further down. This pattern repeats in the MVC cases, and some of these have even lower pressure where the vortex ring around the inflow jet is expected. The rotation visualization in Figures 6A,B, 7A,B confirm that the rotation magnitude is indeed large in those parts. The high strain in the apical part of the ventricle also corresponds well to the high pressure in the same area. A related interesting feature of the rotation and strain is that areas with high rotation often tend to correspond to low strain, and vice versa. An exception to this observation is within the jets, where rotation is 0 and strain is also relatively low. See e.g. Figures 6B,C.

**FIGURE 9:**
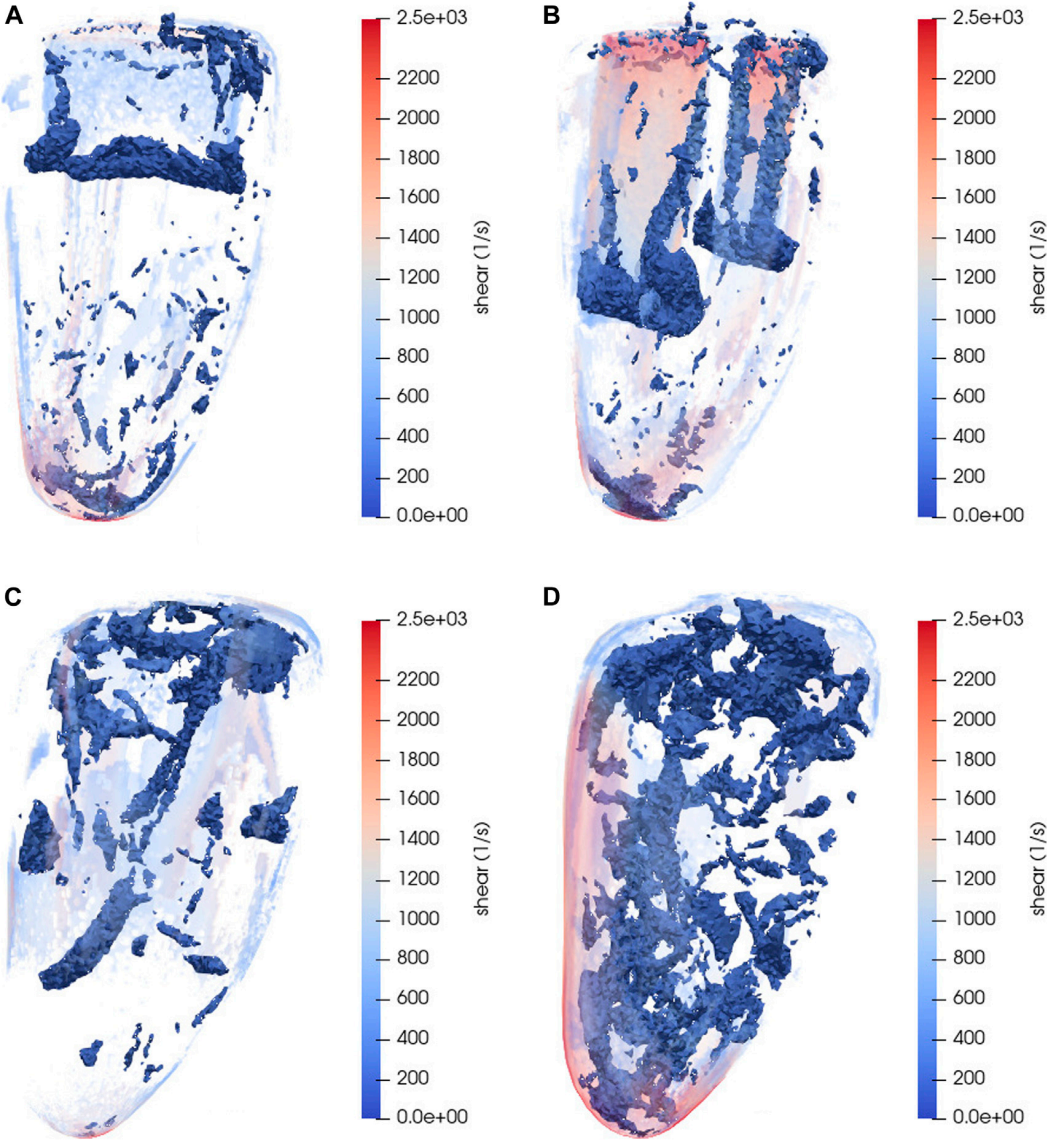
Snapshots of 3D flow structures in the LV blood flow, as determined by the rotation magnitude. The opaque blue structures are all cells that have a rotation magnitude greater than 80 s^−1^. The transparent structures are cells with shear magnitude greater than 500 s^−1^, colored in accordance with the color bars. **(A)** Untreated case, *t* = 0.45 s **(B)** MVC case, *s* = 0.2, *t* = 0.45 s **(C)** Untreated case, *t* = 0.71 s **(D)** MVC case, *s* = 0.2, *t* = 0.71 s. Corresponding 2D snapshots are displayed in Figures 6, 7.

To get a better idea of some of the 3D structures that appear in the LV flow, and how they develop over time, Figure 9 displays snapshots of some 3D structures defined by the computed rotation. The opaque blue structures in the figure mark all cells with rotation magnitude greater than 80 s^−1^, and the transparent multicolored structures are all cells with shear magnitude greater than 500 s^−1^, with values corresponding to the color bars. Both the untreated and MVC case display clear vortex rings forming around the fronts of the jets as connected structures with rotation magnitude greater than a threshold value of 80 s^−1^ (Figures 9A,B). Above these the shear structures are visible at the border around the jet, with significantly higher magnitude in the MVC case. In the rest of the ventricle at this point in time both simulation cases display relatively little rotation, with only small connected volumes reaching above the threshold.

At *t* = 0.71 s, when the E-wave jets have decomposed, Figures 9C,D gives a qualitative view of the distribution of high rotation and shear structures throughout the ventricle. In the untreated case (Figure 9C), the rotation appears as connected features, but with very little in the apical part of the ventricle. The shear is most prominent along the wall. In the clipped case (Figure 9D), the rotation structures appear more numerous, and throughout the entire ventricle in different sizes. The shear is again concentrated on the wall, with higher values as indicated by the color bar. Interestingly, Figure 5 at this point in time indicates that the total amount of rotation/shear magnitudes are similar in both the untreated and clipped (*s* = 0.2) case. We have not investigated this further, but the reason could be that the more intense patterns visible in the MVC case make up a relatively small part of the entire ventricle volume, and that the integrated values in Figure 5 are thus more strongly influenced by large regions of smaller values.

The simulated velocity in the untreated case (Figure 3) is within a reasonable range of a healthy heart, but the peak velocities in the clipped cases may be indicative of mitral stenosis Otto et al. (2021). The opening areas of the clipped cases (Table 2) are also quite small, and MVC treatment would likely not be clinically considered to mitigate regurgitation if the resulting areas were so small. However, in the context of this study, where the MVC intervention provides a simulation case which alters the geometry of the inflow profile of the MV, and thereby also the structure of the intraventricular blood flow and the mechanical stimuli on blood cells, the MVC simulation case fulfills its purpose. Increases in inflow velocity as well as the triple decomposition components in the LV following MVC treatment are still likely to appear in clinically realistic cases due to decreased MV area.

### 4.2 Turbulence

From Figure 9 the differences between the distributions of high rotation and shear structures at different points in time is evident. During the E-wave, much is centered around the jets. Later in diastole, there is a wider distribution over the ventricle. As stated earlier, the Reynolds number during the E-wave is on the scale of 10^4^. Later in diastole, when the initial inflow jet is weakened and the flow breaks down into smaller, more turbulent structures, the velocity is lower. However, using the full width of the ventricle as length scale keeps the Reynolds number approximately unchanged from the jet dominated flow during the E-wave. This corresponds to a Kolmogorov scale of *η* ≈ 10^–4^. Thus our average mesh cell size of 7.2·10^–4^ is close to the Kolmogorov scale, but not quite small enough to resolve all turbulent structures. What we fail to resolve perfectly is modelled as disspiation, using a large eddy simulation (LES) with an implicit turbulence model through the numerical stabilization, an approach which we have developed over the years in different settings, see e.g. Hoffman and Johnson (2006); Bazilevs et al. (2007); Hoffman et al. (2015). The dynamic viscosity used here (*µ* = 2.7·10^–3^ Pa·s, taken from Di Martino et al. (2001)) is on the lower side of typical blood viscosities. Simulating with a higher viscosity would thus not be unreasonable, and this would reduce the Reynolds number and increase the Kolmogorov scale. Studying small scale turbulent structures in detail with the triple decomposition would be interesting, but given the above reasoning this is left for future studies.

### 4.3 Shear-Induced Platelet Activation

Since high shear is known to contribute to thrombosis formation, being able to quantify this in the interventricular blood flow is desirable. Our results suggest that the locations of high shear structures in diastole are similar before and after MVC treatment. They appear mainly around the inflow jets and along the ventricle walls. However, after MVC treatment the magnitude of the shear is higher, possibly as a result of the higher inflow velocities caused by the smaller MV opening areas. As seen in Figure 5, the integrated magnitude over the ventricle is higher for all MVC cases than for the untreated case throughout most of the E-wave and diastasis. This implies that the risk of platelets experiencing higher shear which could lead to thrombosis formation is increased after MVC treatment, but further studies would be required to quantify this risk.

Regarding the high shear along the ventricle walls, it is important to note some uncertainties in this model. There are two major aspects that could contribute to higher or lower magnitudes: surface friction (effectively boundary conditions) and trabeculae carneae. In these simulations, the boundary condition on the ventricle wall is a no-slip condition, which naturally contributes to higher shear rates if the flow velocity close to the wall is high. There is no local mesh refinement close to the wall in this model, and we have also not attempted simulations with other endocardial boundary conditions. When it comes to trabeculae, their effects on the intraventricular blood flow, and how to capture this in simulations, are somewhat unclear. TTE images, such as the ones used to generate the LV geometry here, don’t have high enough resolution to resolve the trabecular structure. A study by Sacco et al. (2018) suggests that the structures decrease wall shear stress, and that this effect could be modelled by adding a porous layer along the endocardial wall. A no-slip boundary condition results in a sharp boundary layer near the ventricle wall, and any relaxation of this boundary condition would likely result in reduced stress near the wall. But further studies are needed to verify this conjecture.

While shear has been clearly linked to thrombosis formation, the case is more open for strain. Our results indicate that the locations of strain structures, like shear, do not change much in the LV after MVC treatment. But as for shear, strain is higher after treatment, both in maximum magnitude and integrated over the ventricle during the E-wave and diastasis.

### 4.4 Improvements to Shear-Induced Platelet Activation Modelling

As previously mentioned, Han et al. (2022) describes models of shear-induced platelet activation which are primarily based on the strain rate tensor originally introduced by Apel et al. (2001) and Bludszuweit (1995), and refined by Faghih and Sharp (2016). However, defining SSS this way, based on the symmetric train rate tensor, does not give an accurate estimate of the shear component of the flow, since components of strain are also included. This is evident both from the symmetric terms in Eq. 11, and from Figure 8, where von Mises-like SSS is visualized alongside components of pure shear, pure strain, and shear and strain summed, as computed by the triple decomposition. Since shear but not strain has been clearly linked to platelet activation, the triple decomposition offers a method to improve current platelet activation models by using only the shear component without strain contamination.

The distinction is important both in terms of magnitude of mechanical stimuli on the blood cells, and in terms of locality. Figure 5 shows that the average magnitudes of strain in the LV are higher than the strain throughout the heartbeat cycle, making shear the dominant factor of SSS. This is qualitatively confirmed by Figure 8 as well. However, the contribution of the strain to SSS is by no means insignificant. In systole, the average strain is around 40–50 s^−1^ in all simulated cases, whereas the shear is mostly in the range of 100–150 s^−1^. In diastole, the average strain peaks in the range of 80–100 s^−1^ for all cases, whereas shear peaks in the range of 350–450 s^−1^. The shear is thus significantly higher, but strain is not insignificant in comparison. Excluding it from shear-induced platelet activation models would thus provide a desirable refinement of results.

As noted, shear and strain also differ in locality in the LV. In Figures 7A,C it was noted that the shear is predominantly large along the edges of the inflow jets in early diastole, whereas the areas of high strain are found near the front of the jets. This is again seen in Figure 8, where it highlights another important difference between the shear and SSS. Since platelet activation depends not only on the magnitude of the shear, but also on the duration of the elevated shear levels the cells are subjected to, it is important to distinguish areas with high potential for platelet activation. The red ellipses in Figure 8 highlight an area where strain is relatively large, and where shear-induced platelet activation computed with SSS may thus be overestimated.

In diastasis the distribution of shear is also quite different from that of strain, as seen in Figure 7D,F, which could also introduce uncertainties in shear-induced platelet activation models.

## 5 Conclusion

Links between shear flow and platelet activation are well established, but models attempting to estimate shear-induced platelet activation are commonly based on measures of shear that also contain strain components. Here we have introduced the triple decomposition of the velocity gradient tensor into a bio-mechanical setting, which allows for more accurate estimation of shear in blood flow. We have demonstrated how the triple decomposition can be used to compute shear in intraventricular blood flow, how it differs from conventional von Mises-like SSS based methods, and how it captures differences in intraventricular mechanical stimuli on blood cells when the blood flow is disrupted by MVC intervention. The relative increases of each individual flow modality following simulated MVC treatment have been presented. The triple decomposition of the velocity gradient tensor opens up for more reliable modelling of shear-induced platelet activation, and insights that may contribute to better understanding thrombosis events.

## Data Availability

The raw data supporting the conclusions of this article will be made available by the authors, without undue reservation.
